# Grid Based Spherical CNN for Object Detection from Panoramic Images

**DOI:** 10.3390/s19112622

**Published:** 2019-06-09

**Authors:** Dawen Yu, Shunping Ji

**Affiliations:** School of Remote Sensing and Information Engineering, Wuhan University, Wuhan 430079, China; yudawen@whu.edu.cn

**Keywords:** spherical convolution, panoramic image, object detection, rotation invariance

## Abstract

Recently proposed spherical convolutional neural networks (SCNNs) have shown advantages over conventional planar CNNs on classifying spherical images. However, two factors hamper their application in an objection detection task. First, a convolution in S2 (a two-dimensional sphere in three-dimensional space) or SO(3) (three-dimensional special orthogonal group) space results in the loss of an object’s location. Second, overlarge bandwidth is required to preserve a small object’s information on a sphere because the S2/SO(3) convolution must be performed on the whole sphere, instead of a local image patch. In this study, we propose a novel grid-based spherical CNN (G-SCNN) for detecting objects from spherical images. According to input bandwidth, a sphere image is transformed to a conformal grid map to be the input of the S2/SO3 convolution, and an object’s bounding box is scaled to cover an adequate area of the grid map. This solves the second problem. For the first problem, we utilize a planar region proposal network (RPN) with a data augmentation strategy that increases rotation invariance. We have also created a dataset including 600 street view panoramic images captured from a vehicle-borne panoramic camera. The dataset contains 5636 objects of interest annotated with class and bounding box and is named as WHU (Wuhan University) panoramic dataset. Results on the dataset proved our grid-based method is extremely better than the original SCNN in detecting objects from spherical images, and it outperformed several mainstream object detection networks, such as Faster R-CNN and SSD.

## 1. Introduction

A vision-based object detection task is to recognize and locate objects of interest in a given image efficiently and accurately. Convolutional neural networks (CNNs) have shown outstanding performances in object detection [1,2,3], as well as in other vision tasks such as image classification [4,5,6] and semantic segmentation [7,8,9]. Although many CNN based approaches have obtained satisfactory results on detecting objects in planar images [5,10,11], their applications on other manifolds, such as sphere, are basically unexplored. As omnidirectional or panoramic camera has shown a wide range of applications in virtual reality [12], driverless cars [13], monitoring systems [14] and SLAM [15,16], how to detect objects from a spherical image becomes more significant. 

Different from planar images, position-related distortions are unavoidable when projecting a spherical signal to its planar representation. This type of distortion was deeply investigated in Gauss’s age for mapping the Earth. In this deep learning age, a planar CNN must depend on the translation invariance of convolutions to obtain its power. This prerequisite cannot be met when using a spherical image because the space of moves on a sphere is a set of 3D rotations, i.e., a special orthogonal group called SO(3) instead of 2D translations. 

Most recently, two novel works proposed spherical CNN (SCNN), which defined convolution in the SO(3) space instead of the 2D plane to strictly preserve rotation invariance [17,18]. This kind of SCNN has shown obvious advantages over conventional CNNs in spherical image classification, for example, classifying digits in a spherical image simulated from the MNIST dataset and labelling 3D objects from the ShapeNet dataset [19]. However, there are two critical restrictions when extending a classification task to object detection.

The first one is that the spherical convolution in the SO(3) space causes the accurate location information of an object’s signal in the sphere space to be lost. There is no simple way to retrieve the object’s bounding box. 

The second one is that the bandwidth (corresponding to pixel resolution in a planar image) should be wide enough to preserve a small object’s signal, because the spherical convolution is performed on the whole sphere, instead of a local image patch. Although [17] reported that using a SCNN with fewer parameters could obtain better classification results than a conventional CNN in classifying rotated spherical images, they utilized a very small spherical image (64 × 64) to alleviate the bandwidth burden. Whereas, in object detection, much larger images may have to be handled. Furthermore, an object of interest only covers a small part of the whole sphere, in most cases. 

**Figure 1:**
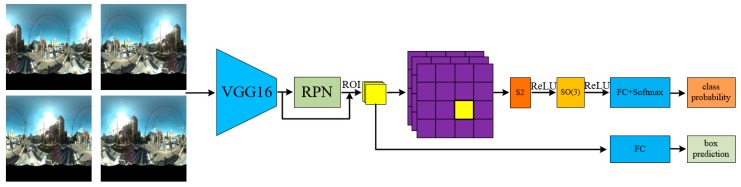
The workflow of our grid based spherical CNN (G-SCNN) for object detection. The omnidirectional images with rotation-invariance augmentation are fed into the VGG-16 to extract features. The RPN is used to provide bounding box proposals. The candidate objects in each bounding box are projected onto a grid map, which is then classified by the SCNN. The accurate bounding box is also retrieved.

In this study, we present a grid based SCNN (G-SCNN) for detecting objects in panoramic images (Figure 1). Our method extends the applications of SCNNs to object detection for the first time. The main idea is as follows: We unwrap a sphere to a conformal grid map (e.g., 28 × 28) to be the input of the network according to a given input bandwidth and then scale the feature maps of an object to cover a certain area of the grid map (e.g., 7 × 7), instead of using their original size. The strategy largely solves the bandwidth problem in an efficient way and guarantees successful applications of a SCNN to object detection. We also partially solved the problem of location information loss by using a rotation augmented planar region proposal network (RPN).

The main contributions of the paper are the following:

(1) We extend the SCNN’s capacity to object detection via a simple and effective method. The object detection results are extremely better than the original SCNN and outperformed mainstream object detection methods, such as Faster R-CNN and SSD.
(2) An omnidirectional image dataset (http://study.rsgis.whu.edu.cn/pages/download/) of real street scenes with multi-class annotations is created for assessing object detection algorithm, which is a beneficial supplement to those simulated spherical image datasets and indoor datasets.

## 2. Related Work

There are numerous algorithms developed for detecting objects in planar images, from early classic sliding-window strategies [20,21] to current CNN based approaches [11,22,23,24]. In contrast, methods designed for omnidirectional images are much fewer. In the task of omnidirectional image classification or object detection, the main challenge is that large geometric distortions must be introduced when projecting a spherical signal to the equivalent planar representation.

To mitigate the effects of this location-related distortion, Su et al. [25] used planar convolutions with different kernel sizes at the different rows of a spherical image. Coors et al. [26] designed a network called SphereNet, in which rotation invariance was encoded into planar CNNs by applying a set of pre-designed convolutional filters at different locations. Different from [26], Dai et al. [27] proposed a network to automatically learn adaptive convolutional filters for different locations. Tateno et al. [28] developed a distortion-aware deformable convolution filter to regress depth information from panoramic images. Zhao et al. [29] sampled an irregular grid map based on the pixels’ distortion level and applied square convolutional kernels on the grid map for spherical image classification. Pais et al. [30] used reinforcement learning to predict pedestrians’ positions by projecting the 3D bounding boxes of pedestrians onto panoramic images. There are also methods which only use adequate planar convolutional filters to fit the rotation distortions [31,32].

By introducing graph-based representations, Khasanova et al. [33] gave convolutional filters the ability to respond consistently to a signal despite of its different positions on a sphere. Khasanova et al. [34] further developed a graph-based network, where features are inherently invariant to isometric transformations, such as rotation and translation. Monroy et al. [35] mapped an omnidirectional image to six image patches with fixed view angles to mitigate spherical distortion. Marcos et al. [36] simulated rotation-invariance features by applying each convolutional filter at multiple orientations. In summary, these methods attempt to reduce the impact of spherical projection distortion either through re-sampling the spherical images or through re-sampling the filters.

Very recently, rigorous analytical methods instead of numerical simulation have been developed. Worrall et al. [37] proposed harmonic networks, which achieved both rotation and translation equivariance by replacing planar convolutional filters with circular harmonics. By representing sources of variation with symmetry groups, Gens et al. [38] gave a generalization of CNNs that formed feature maps over arbitrary symmetry groups. Cohen et al. [39] put forward group equivariant CNNs for sphere images through exploring symmetries. Cohen et al. [18] further encoded rotation equivariance into classification networks by performing convolutions in S2 (a two-dimensional sphere in three-dimensional space) and SO(3) space. Esteves et al. [17] modeled 3D data with multivalued spherical functions and proposed a spherical convolution in the spherical harmonic domain for 3D object classification. These methods attempt to treat signals on different positions of a sphere as analytically equivalent, but their applications are limited to the classification and retrieval of spherical images.

To our knowledge, the rigorous spherical convolution in the SO(3) space [17,18] has not been applied to an object detection task.

## 3. Methods

We briefly review the spherical convolution presented in the work of [18], then, we introduce our G-SCNN and its application in object detection.

### 3.1. Spherical Convolution

In three-dimensional Euclidean space, the well-known rotation matrix *R* is a 3 × 3 unit orthogonal matrix. All the matrices constitute a special orthogonal group, SO(3), which can be parameterized by ZYZ-Euler angles with *α* ∈ [0, 2*π*], *β* ∈ [0, *π*], *γ* ∈ [0, 2*π*], or angles with several other rotation sequences. A function *f* defined on SO(3) can, therefore, be written as a function with the three Euler angles as variables [40]. For a point on a sphere, i.e., *x* ∈ S2, the product of *R* and *x* represents the result of rotating *x*. Analogously, a general rotation operator *L*_R_ performs a rotation on the function *f*, as follows:

[*L*_R_*f*](*x*) = *f*(*R*^−1^*x*). (1)

The inner product of spherical signals *ψ* and *f* could be written as follows:

(2) $$\langle \psi ,f\rangle =\int_{s^{2}}\sum_{k=1}^{K}\psi_{k}(x)f_{k}(x)dx.$$

By denoting a spherical point or filter with latitude-longitude coordinate [α, *β*], where *α* ∈ [0, 2π] and *β* ∈ [0, π], the definition of S2 convolution is given as:

(3) $$\left[\psi *f\right](R)=\langle L_{R}\psi ,f\rangle =\int_{S^{2}}\sum_{k=1}^{K}\psi_{K}(R^{-1}x)f_{k}(x)dx.$$

The output of S2 convolution is a function on SO(3) represented by ZYZ-Euler angles. 

The rotation operator on SO(3) space is as follows:

[*L*_R_*f*](*Q*) = *f*(*R*^−1^*Q*), (4)

where *R*, *Q* ∈ SO(3). Analogous to S2 convolution, the SO(3) convolution is expressed as follows:

(5) $$\left[\psi *f\right](R)=\langle L_{R}\psi ,f\rangle =\int_{SO(3)}\sum_{k=1}^{K}\psi_{K}(R^{-1}Q)f_{k}(Q)dQ.$$

As convolutions in an image domain can be equivalently represented as multiplications in Fourier space, a discrete Fourier transform theorem is used for the efficient computation of S2/SO(3) convolutions [18]. The process of a S2/SO(3) convolution is as follows: the spherical signals *f* and *ψ* (in latitude-longitude coordinates) are Fourier transformed, multiplied in frequency domain, summed over channels, and finally inversely Fourier transformed.

### 3.2. Grid-Based Spherical Convolutions

An S2 or SO(3) convolution is the building block of a SCNN for image classification or object detection. The bandwidth of an S2/SO(3) convolution determines the resolution of an input, as well as what level of detail with which it is processed. Taking SO(3) convolution as an example, a continuous signal *f* (*α*, *β*, *γ*) is firstly quantified to a 2*B* × 2*B* × 2*B* cube, as follows:

(6) $$\left\{\left(\alpha_{j1},\beta_{k},\gamma_{j2}\right)|0\leq k,j_{1},j_{2}\leq 2B-1\right\},$$

where *B* is the bandwidth and

$$\alpha_{j1}=\frac{2\pi j_{1}}{2B},\;\beta_{k}=\frac{\pi (2k+1)}{4B},\;\gamma_{j2}=\frac{2\pi j_{2}}{2B}.$$

The discrete SO(3) Fourier transform at bandwidth *B* can be expressed as:

(7) $${\overset{⌢}{f}}_{MM^{\prime }}^{l}={\left(\frac{\pi }{B}\right)}^{2}\sum_{j_{1}=0}^{2B-1}\sum_{j_{2}=0}^{2B-1}\sum_{k=0}^{2B-1}w_{B}\left(k\right)f\left(\alpha_{j1},\beta_{k},\gamma_{j2}\right){\tilde{D}}_{MM^{\prime }}^{l*}\left(\alpha_{j1},\beta_{k},\gamma_{j2}\right),$$

where *w_B_*(*k*) is the quadrature weights associated to the bandwidth *B* [41], ${\tilde{D}}_{MM^{\prime }}^{l}$ is the L2-normalized Wigner *D*-function, and ${\tilde{D}}_{MM^{\prime }}^{l*}$ is its complex conjugate [40]. 

Analogously, a spherical signal, g(θ,ϕ), is quantified to a 2*B* × 2*B* grid, as follows:

(8) $$\left\{\left(\theta_{j},\phi_{k}\right)|0\leq j,k\leq 2B-1\right\},$$

where $\theta_{j}=\frac{\pi (2j+1)}{4B}$, $\phi_{k}=\frac{2\pi k}{2B}$ are the latitude and longitude, respectively.

An output bandwidth, which determines the level of detail of the output features, is also required to set for the inverse Fourier transform in an S2/SO(3) convolution. The output bandwidth must be no greater than the input bandwidth.

A typical SCNN for image classification consists of an S2 convolution that translates input to SO(3) feature maps and a series of SO(3) convolutions that learn higher semantic features with rotation invariance. In the work of [18], an input bandwidth of 30 was used for classifying digits from 60 × 60 spherical images, which exactly preserves the original resolution. An output bandwidth of 10 of the S2 convolution was used to ensure the information is not over compressed. Additionally, the digits almost covered the whole image. Hence, the bandwidth quantization and compression have no impact on learning satisfactory representations. 

However, in an object detection task, the images are much larger, but the bandwidth cannot be set as large as them. For example, a single SO(3) convolution with an input and output bandwidth of 256 would occupy several gigabytes [40]. With a commonly used 6G GPU, the maximum output bandwidth just reaches 32, even though only one S2 and one SO(3) convolution layer are used in a simple classification or object detection network. 

**Figure 2:**
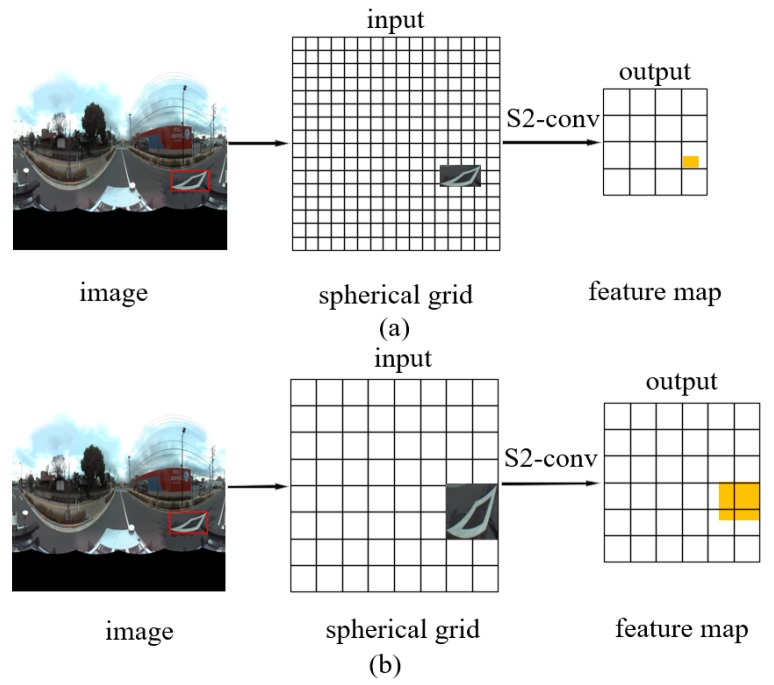
The original spherical convolution (**a**) and our grid-based spherical convolution (**b**).

Figure 2a shows an object (in the red box) in a square image with width *W*. The object is projected onto the quantized 2*B*_1_ × 2*B*_1_ grid, where the input bandwidth *B*_1_ is set to W/2. By setting an available output bandwidth *B*_2_ (typically *B*_2_ << *B*_1_), the S2 convolution outputs a very small and over-compressed feature (the yellow block). Hence the S2/SO(3) convolutions hardly learn any effective representation of the object. This forbids a successful application of the original SCNN in object detection. 

Figure 2b shows the grid-based spherical convolution we proposed. The key rule is keeping the output bandwidth close to the input bandwidth, to avoid over-compression, and ensuring the object or its feature map covers an adequate space on the input grid map. We scaled the object to exactly cover *m* × *m* grids before feed it into the S2 convolution. The number *m*, the input bandwidth *B*_1_, and the output bandwidth *B*_2_ are set according to the above rule and the available GPU memory. In this study, we set 2*m = B*_1_ = 14 and *B*_2_ = 10 in the S2 convolution, and the bounding box of an object was scaled to cover 1/16 of the grid map.

### 3.3. G-SCNN for Object Detection

After S2 or SO(3) convolutions the bounding box of an object in the spherical space is largely lost and there is no easy way to retrieve it. In addition, how to find a feature’s bounding box in the SO(3) space has not yet been explored. In this study, we used a simulated manner that resamples signals from multiple viewpoints to increase rotation invariance. Then, the object’s bounding box was detected by a conventional planar regional proposal network (RPN) [5]. One input image was resampled to 8 images at each 45° rotation angle around the Z axis, pointing upwards. In other words, a sphere is unfolded 8 times at different horizontal directions, as follows: (θ=0,ϕ=kπ/4) where *k* = 0, 1, …, 7. This is suitable because most of the interested objects in street-view omnidirectional images lie approximately along the 0° latitude line.

**Figure 3:**
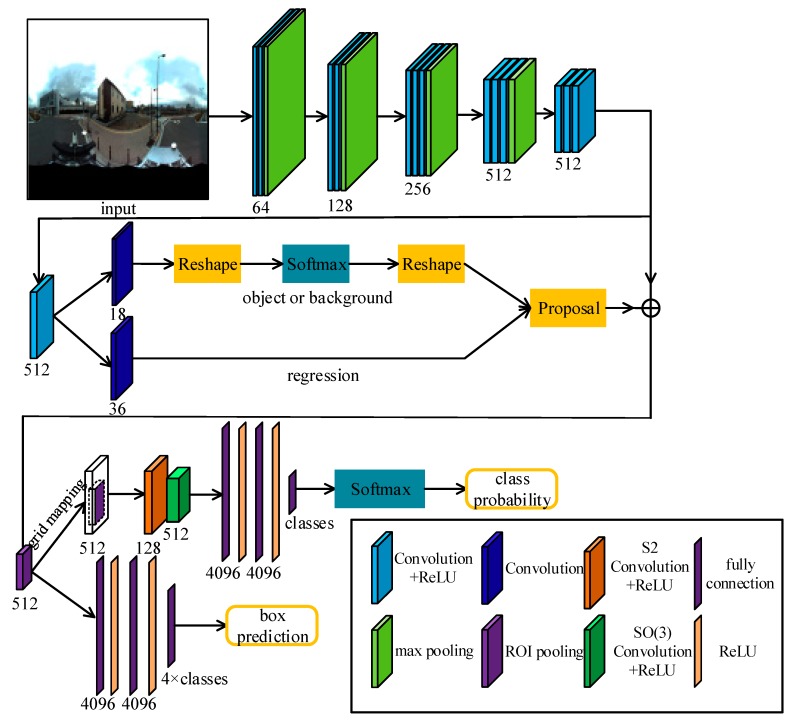
Our G-SCNN for object detection. Omnidirectional images with rotation augmentation are fed into the network for detecting objects of interest.

In Figure 3, the RPN with a VGG-16 backbone is utilized to detect bounding boxes. For each box, the corresponding features in the Conv5-3 layer (the last convolutional layer of the VGG-16) were resampled to 7 × 7 to feed the grid based spherical convolution (Figure 2b). The SCNN for object detection consists of one S2 convolution and one SO(3) convolution, followed by three fully connected (FC) layers and a softmax, i.e., S2-ReLU-SO(3)-ReLU-FC×3-softmax.

### 3.4. Data Preprocessing

Our panoramic images were collected from a multiple fisheye camera rig. First, the six fisheye images were projected onto a sphere according to the calibration parameters between the fisheye cameras and the virtual panoramic camera and a given sphere radius *r*, as follows:

(9) $$X=mR_{i}K_{i}(x)+T_{i},$$

(10) $$\left\|X\right\|=r^{2}.$$

In Equation (9), ***T****_i_* and ***R****_i_* are the translation vector and rotation matrix between the *i*-th fisheye camera and the panoramic camera. The calibration model *K*(·) projects the fisheye image to an equivalent planar image. Here, we use a generic fisheye calibration model, proposed in [42].

Second, the spherical point ***X*** = [*X Y Z*]^T^ is projected to a Driscoll–Healy image by using Equations (11) and (12). Each pixel (*x*′, *y*′) in the Driscoll–Healy grid exactly corresponds to the spherical signal (S2) according to *α* = 2π*x*′/2*B* and *β* = π*y*′/2*B*, where *α* ∈ [0, 2*π*], *β* ∈ [0, *π*]. 

(11) $$\varphi_{h}=\arccos\left(\frac{Y}{X}\right),\varphi_{v}=\arccos\left(\frac{Z}{\sqrt{X^{2}+Y^{2}+Z^{2}}}\right).$$

(12) $$x^{\prime }=\frac{2\varphi_{h}b}{2\pi },y^{\prime }=\frac{2\varphi_{v}b}{\pi }.$$

## 4. Experiment and Analysis

### 4.1. Dataset

Some existing indoor datasets, such as the Matterport3d [43] and ScanNet [44], may be used to generate spherical panoramas, but the process is complex and not rigorous. Therefore, they were rarely used in the studies of spherical object detection. Other studies [25,26] used semi-synthetic or synthetic datasets, but it is more valuable to use real data for testing.

**Figure 4:**
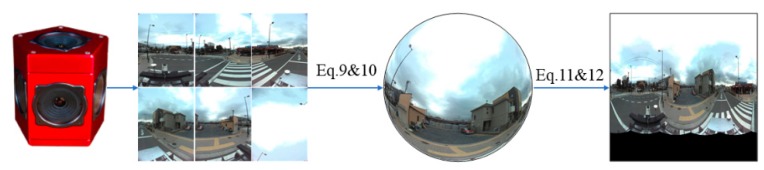
Producing Driscoll–Healy image from a multi-camera rig.

Due to the lack of real omnidirectional image datasets with the annotations of objects’ bounding box, we created a street scene dataset called WHU (Wuhan University) panoramic dataset. The dataset consists of 600 RGB images captured from vehicle-borne PGR’s Ladybug3 camera [45] in Kashiwa and Dagong cities, Japan. The camera consists of six fisheye lenses, each of which has a maximum 1616 × 1232 pixel resolution. The focal length of the fisheye camera is 3.3 mm and the radius of the panoramic sphere is set to 20 m. After the data preprocessing (Figure 4), four classes of objects of interest, including light, crosswalk, crosswalk warning line (a diamond sign indicating the upcoming crosswalk), and car were manually labelled in the Driscoll–Healy images (see Table 1). Cross-checking was carefully carried out to minimize the risk of false judgement. In object detection, one third of the data is used for training and the rest for testing. All experiments are executed on a Linux PC with an Intel i5-8400 CPU, a GeForce GTX 1080 TI 11G GPU, and 8G memory.

### 4.2. Classification

Classification tasks using a SCNN were only testified in virtual reprojection of 2D planar images [18]. We checked the SCNN’s capability in real and larger street scene. Five classes of objects, including building, car, crosswalk, crosswalk warning line, and streetlight were selected from 2558 omnidirectional images for testing. For each omnidirectional image, only one interested object with adequate size was reserved and the rest of the pixels were set to zero. We scaled these processed images to 512 × 512 pixels. Both planar and spherical CNNs use light and comparable structures. The planar CNN structure is as follows: Conv-ReLU-Conv-ReLU-FC-Softmax, with 5 × 5 kernel size, 32, 64 channels, and 443 k parameters in total. The spherical CNN structure is as follows: S2-ReLU-SO(3)-ReLU-FC-Softmax, with 256, 24 and 12 bandwidth, 32, 64 channels and 149 k parameters. We trained both networks for 100 epochs using an ADAM optimizer with a learning rate of 10^−4^ and a batch size of 32.

In the first experiment, two third of the 2558 samples were randomly selected for training and the rest for testing. In the second experiment, those objects located on the left sphere (1427 samples) were selected for training and the objects on the right sphere (1131 samples) were used for testing. Table 2 shows that, when using the spherical CNN, the classification accuracy is improved by 4.8% in the first experiment and dramatically improved about 70% in the second experiment, compared to the planar CNN, respectively. This proved the effectiveness and advantage of a spherical CNN for classifying spherical images.

### 4.3. Object Detection

The experiments were designed to evaluate object detection performance in our spherical street-view dataset. Six methods, the Faster R-CNN [5], Faster R-CNN with FPN (short as FPN) [46], SCNN [18], SSD [47], our G-SCNN, and a variant of the Faster R-CNN (named as Faster R-CNN+) were used for comparison. All of them share the same planar RPN strategy and 600 × 600 image inputs. In all tests, the batch size was set to 1. The size of S2 filters was set to 24 points, which is comparable to a planar convolution with a kernel size of 5 (i.e., 25 points). In the SO(3) convolution, a third dimension was introduced, wherein we increased the number of filter points to 72. Each network was separately trained for 70,000 iterations, with the SGD optimizer starting with an initial learning rate of 10^−3^, which was decreased by a factor of 10 after 50,000 iterations.

The SCNN we used is similar to the structure used for classification [18]. After ROIs are detected by using the RPN, each ROI image patch in the input image is projected onto a blank 600 × 600 grid map (i.e., bandwidth = 300), which is then fed into the SCNN structure, as follows: S2-ReLU-SO(3)-ReLU-SO(3)-ReLU-FC×3-softmax. The input and output bandwidths are 300 and 18 in S2 convolution, 18 and 12 in the first SO(3) convolution, and 12 and 8 in the second SO(3) convolution. The number of output channels of the three convolutions is 24, 48, and 96, respectively.

In our G-SCNN, the ROI is retrieved from the 16× down-sampled features (Conv5-3) of the VGG-16 and resampled to 7 × 7 after ROIPooling. It is then projected onto a 28 × 28 grid map (bandwidth = 14) by keeping the same location and resolution. The grid map is then fed into our spherical CNN: S2-ReLU-SO(3)-ReLU-FC×3-softmax, where the input and output bandwidths are 14 and 8 in the S2 convolution and 8 and 6 in the SO(3) convolution respectively. The numbers of input and output channels of the S2 convolution are 512 and 128, respectively. The number of output channels of the SO(3) convolution is 512.

We use the Faster R-CNN+ for ablation experiments, which has the same configuration with our G-SCNN, except the spherical convolutions are replaced with planar convolutions. We keep the output size of the planar convolutions as 28 × 28 and use a kernel size of 5 × 5 to coincide with the S2 and SO(3) convolutions.

For the SSD, the input images were scaled to 512 × 512 pixels, due to the structure of the SSD.

Table 3 shows the object detection results using the Faster R-CNN, FPN, Faster R-CNN+, SCNN, SSD, and our G-SCNN methods, respectively. The AP (average precision) is counted on IoU > 0.5. The SCNN performs the worst and 25% lower than the other methods on mAP (mean average precision), demonstrating the incompetence of applying an original SCNN for object detection. The Faster R-CNN has reached 57.9% mAP. The mAP of the Faster R-CNN+ is 2.5% lower than ours, which demonstrates that the higher performance of our G-SCNN is completely due to the introduction of the specific spherical convolutions. The mAP of the SSD is 3.3% lower than ours and the mAP of FPN is close to ours. 

Table 4 shows the object detection results on AP_75_ (IoU > 0.75). The mAP of all the methods dropped and our method outperformed the second-best Faster R-CNN 2.8%. The mAP of the SSD and the FPN dropped more. Compared to the results of Table 3, it indicates that the bounding box accuracy of our method is also better than that of the other methods. 

Table 5 shows the object detection results with data augmentation. Each training image was resampled to 8 images at each 45° horizontal interval. We performed offline augmentation on these images and fed them into the network for training. The Faster R-CNN, Faster R-CNN+, FPN, and our method got 5.7%, 3.6%, 4.2%, and 4.1% improvements on mAP_50_, and 5.0%, 2.2%, 2.8%, and 5.3% improvements on mAP_75_, respectively. The rotation sampling increases the planar CNN’s capacity for detecting rotated objects, however, our method still performs the best on both mAP_50_ and mAP_75_. The performance of the SCNN got no improvement because its key restriction is lacking enough bandwidth for objects of interest. The performance of the SSD also showed almost no improvement as it is the only method that does not use an RPN for searching bounding boxes.

Table 6 shows the results of applying the rotation augmented models on the rotated test data. One test image was resampled 8 times and the test data now go 8× larger. The mAP of all the methods dropped a little. On mAP_50_ the Faster R-CNN dropped 4%, the FPN dropped 3%, and our method dropped 2%. Our method outperformed the second best methods 1.4% and 2.5% on mAP_50_ and mAP_75_ respectively, and showed better rotation invariance. The SCNN performed the worst.

**Figure 5:**
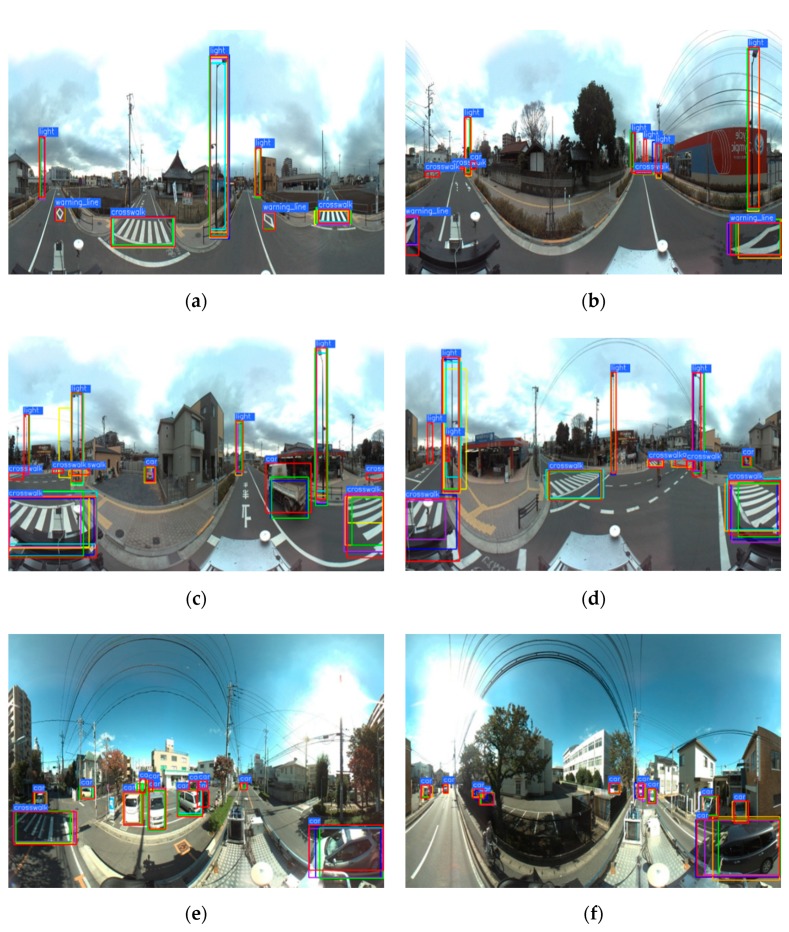
Examples of detected objects of interest with different methods. Red, blue, green, purple, yellow, orange, and cyan boxes are ground truth, the results of (**a**) our method, (**b**) the Faster R-CNN, (**c**) the Faster R-CNN+, (**d**) the spherical CNN, (**e**) the FPN, and (**f**) SSD, respectively.

Figure 5 shows some examples of detected objects in the spherical images. Our G-SCNN (blue box) detected most of the objects of interest (the ground truth is denoted with red box). The bounding boxes of our method are closer to the ground truth than that of the other methods, for example, the crosswalk warning line in Figure 5b, the crosswalks in Figure 5c,d, and the cars in the right side of Figure 5f. The original SCNN (yellow box) only detected a few objects with obvious false negatives. The Faster R-CNN (green box) and Faster R-CNN+ (purple box) could also detect most of the objects. However, for those large and distorted objects, like crosswalk warning line and crosswalk, they performed worse than our method and their bounding boxes were less accurate.

## 5. Discussion

### 5.1. Detecting Small Objects

A small object in a big spherical image causes difficulty in both classification and object detection, when using spherical convolution. Taking traffic signs as an example, we checked the performances of the six methods for detecting small objects from the street-view omnidirectional images. A total of 578 traffic signs in the 600 omnidirectional images were manually labeled. The average area of traffic signs in our dataset is 173.3 pixels and most of them cover less than 0.1% of the area of the 600 × 600 image. According to the classification criteria of the MS-COCO dataset [48], there are 573 small traffic signs (area ≤ 32 × 32 pixels), 5 medium traffic signs (32 × 32 pixels < area ≤ 96 × 96 pixels) and zero large traffic signs (area > 96 × 96 pixels). With one third of samples for training and the rest for testing, the performances of the six methods on mAP_50_ are listed in Table 7. Compared to the other objects in Table 3, the mAP_50_ of almost all the methods dropped about 30%, and the mAP_50_ of the SCNN decreased to below 10%. The Faster R-CNN and FPN were shown to be slightly better than our method as the rotation deformation of a small object could be very slight.

### 5.2. Efficiency

Table 8 shows the training efficiency of the six methods. The classic CNN methods are much faster, since they only perform convolutions in planar space. Although the FFT is utilized, the spherical CNN based methods with S2/SO(3) convolutions in SO(3) space show relatively lower efficiency. We investigated why our method is slower than the original SCNN. We found the SCNN basically missed those small objects and, therefore, converged to a result on only a part of the training samples. Our method showed a normal efficiency of spherical CNNs for object detection. 

### 5.3. Features in SO(3) Space

**Figure 6:**
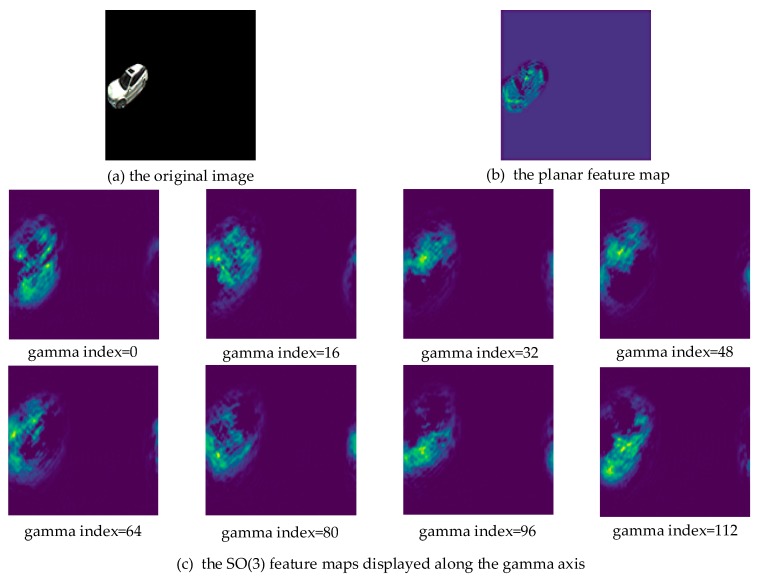
A car in a Driscoll-Healy image (**a**), (**b**) and (**c**) correspond to the car’s feature maps of planar convolutions and S2 + SO(3) convolutions, respectively.

The features of spherical convolutions encapsulate the information of a signal on arbitrary positions of the sphere. An SO(3) convolution realizes this by rotating the filters. Figure 6a shows a car in a 128 × 128 Driscoll–Healy image (bandwidth *B* = 64). Figure 6b,c visually exhibits the car’s feature maps after two planar convolutions and S2 + SO(3) convolutions, respectively. Note that we kept the size of the feature maps the same as the input. An SO(3) feature is a 3-dimensional tensor with the size of [2 × *B*, 2 × *B*, 2 × *B*], which is different from a planar feature (a 2-dimensional tensor with the size of [2 × *B*, 2 × *B*]). Therefore, we display the SO(3) feature maps at equal intervals along the *γ* axis in Figure 6c.

Figure 6b shows that the position of the planar feature exactly corresponds to the original image. However, the position of the spherical features does not. The rotations of the filters caused the output features to rotate along the *γ* axis, losing accurate location information. We tried summing the SO(3) signal over the gamma component to retrieve the corresponding signal on S2. However, we did not obtain accurate bounding boxes of interested objects and the object detection network did not converge. How to accurately locate objects in SO(3) space has not been explored and we hope to discover an effective way in future.

### 5.4. The Impact of Encoder

The performances of the popular structures, the VGG16, VGG19, ResNet50, ResNet101, and ResNet152, were respectively evaluated as the encoder of the RPN in our G-SCNN. All of the five networks shared the same settings in training and the test. In Table 9, the ResNet152 and VGG16 obtained the highest mAP_50_ score, whereas the VGG16 obtained the highest mAP_75_ score. Considering both the accuracy and the efficiency, the VGG16 is the best structure for our object detection task from spherical images.

## 6. Conclusions

This study proposed a novel and effective grid based spherical CNN (G-SCNN) that extends the capacity of a spherical CNN to object detection for the first time. The experiments have proved our method conquers the shortcomings of the original SCNN, i.e., lacking of enough bandwidth, through introducing a grid map before the S2 convolution. The grid map keeps the output bandwidth close to the input bandwidth, and ensures the objects’ information is effective in the S2/SO3 convolutions performed on the whole spherical images. The G-SCNN also outperformed several mainstream CNN based object detection methods, both on mAP_50_ and mAP_75_. Additionally, we created an open street-view panoramic image dataset with multi-class annotations for object detection, which is a beneficial supplement to existing simulated spherical image datasets and indoor datasets. 

As it is the first attempt, the current spherical CNN structure is still subject to the planar RPN for finding bounding boxes. More sophisticated algorithms and structures might be further explored for directly locating bounding boxes in the SO3 space.

## Figures and Tables

**Table 1:** The spherical street-view dataset containing 600 images and 5058 objects.

Category	Light	Crosswalk	Warning Line	Car	Total
Number	1777	867	355	2059	5058

**Table 2:** Classification results with spherical and planar CNNs.

Method	Spherical CNN	Planar CNN
random	93.2%	88.4%
left: right	86.7%	18.5%

**Table 3:** Object detection results of different methods on mAP_50_.

Method	Light	Crosswalk	W-Line	Car	mAP_50_
Faster	0.641	0.448	0.731	0.498	0.579
FPN	0.619	0.504	0.631	**0.622**	0.594
Faster+	0.622	0.441	0.726	0.513	0.575
SCNN	0.464	0.383	0.256	0.254	0.339
SSD	0.529	**0.553**	0.702	0.485	0.567
ours	**0.678**	0.446	**0.779**	0.497	**0.600**

**Table 4:** Object detection results of different methods on mAP_75_.

Method	Light	Crosswalk	W-Line	Car	mAP_75_
Faster	0.358	0.184	0.491	0.212	0.311
FPN	0.329	0.150	0.337	0.190	0.251
Faster+	0.366	0.178	0.486	0.194	0.306
SCNN	0.282	0.197	0.161	0.125	0.192
SSD	0.260	0.208	0.328	0.186	0.245
ours	**0.391**	**0.231**	**0.512**	**0.221**	**0.339**

**Table 5:** Object detection results of different methods trained with rotation augmented samples. The accuracy of single class is counted on AP_50_.

Method	Light	Crosswalk	W-Line	Car	mAP_50_	mAP_75_
Faster	0.702	0.518	**0.810**	0.517	0.636	0.361
FPN	0.622	0.525	0.720	**0.679**	0.636	0.279
Faster+	0.696	0.485	0.744	0.519	0.611	0.328
SCNN	0.494	0.395	0.213	0.257	0.340	0.193
SSD	0.563	**0.566**	0.676	0.514	0.579	0.247
ours	**0.718**	0.521	0.803	0.526	**0.641**	**0.392**

**Table 6:** Applying the rotation augmented models on the rotated test data. The accuracy of single class is counted on AP_50_.

Method	Light	Crosswalk	W-Line	Car	mAP_50_	mAP_75_
Faster	0.702	0.450	0.723	0.513	0.597	0.344
FPN	0.598	0.517	0.710	**0.601**	0.606	0.266
Faster+	0.690	0.440	0.721	0.516	0.592	0.316
SCNN	0.480	0.390	0.202	0.248	0.330	0.182
SSD	0.556	**0.556**	0.664	0.506	0.570	0.244
ours	**0.712**	0.520	**0.729**	0.518	**0.620**	**0.369**

**Table 7:** Detecting small traffic signs in spherical street-view images. The accuracy is counted on mAP_50_.

Method	Faster R-CNN	FPN	Faster R-CNN+	SCNN	SSD	Ours
No augment	0.1988	**0.232**	0.1749	0.0991	0.1717	0.1762
With augment	0.2531	**0.322**	0.2072	0.0825	0.1761	0.2421

**Table 8:** Training efficiency of different methods.

Method	Train Time (h)
Faster R-CNN	2.9
Faster R-CNN+	4.3
SCNN	14.9
SSD	2.5
FPN	6.5
Ours	32.6

**Table 9:** The test accuracy and training time of different encoders in our G-SCNN.

Feature Extractor	mAP_50_	mAP_75_	Training Time (iter/s)
VGG-16	0.600	**0.338**	**1.67**
VGG-19	0.551	0.282	1.69
ResNet-50	0.574	0.296	3.42
ResNet-101	0.578	0.301	3.47
ResNet-152	**0.602**	0.325	3.50
